# MKI67 as a potential diagnostic biomarker in pulmonary hypertension

**DOI:** 10.3389/fped.2022.1016889

**Published:** 2022-11-01

**Authors:** Huiling Zhou, Ke Gong, Yifeng Yang, Qin Wu, Qiuguo Wang, Yadan Shen, Li Xie, Yibo Gong, Haidan Liu, Jijia Liu

**Affiliations:** ^1^ Department of Cardiovascular Surgery, The Second Xiangya Hospital of Central South University, Central South University, Changsha, China; ^2^ Extracorporeal Life Support Center of Cardiovascular Surgery, The Second Xiangya Hospital of Central South University, Central South University, Changsha, China; ^3^ The Clinical Center for Gene Diagnosis and Therapy of the State Key Laboratory of Medical Genetics, the Second Xiangya Hospital of Central South University, Central South University, Changsha, China

**Keywords:** pulmonary hypertension, right heart failure, RNA-sequence, biomarker, MKI67

## Abstract

**Background:**

Right heart failure results from advanced pulmonary hypertension (PH) and has a poor prognosis. There are few available treatments for right heart failure. Pulmonary artery remodeling, including changes in pulmonary artery endothelial cells to endothelial-mesenchymal cells, and aberrant fibroblast and pulmonary artery smooth muscle cell (PASMC) proliferation, are characteristics of the pathophysiological process of PH. As a result, the clinical situation requires novel PH diagnostic and treatment targets.

**Methods:**

Monocrotaline was used to create an animal model of PH, and lung tissue was removed for transcriptome sequencing. The targets with the highest differences were chosen for transfection after possible targets were identified using bioinformatic techniques and confirmed by qPCR to examine their function in hypoxic PASMCs.

**Results:**

After sequencing 781 differentially expressed mRNAs, we compared them with the GEO dataset and found 43 differentially expressed genes. We chose the top three scores for further study and verification and discovered that MKI67, a crucial element of the cell cycle that regulates PASMC proliferation, had the greatest effect. After suppressing MKI67 in PASMCs, both cell proliferation and migration decreased.

**Conclusion:**

Several potential targets were chosen for this study, and MKI67 was found to play a regulatory role in cell migration and proliferation. This implies that PH can be diagnosed and treated using MKI67.

## Introduction

Rare pulmonary hypertension (PH), which advances to an advanced stage and causes right heart failure (RHF), has a poor prognosis. There are very few therapeutic options available once RHF begins. It is simple for many congenital cardiac conditions to produce PH first, followed by RHF (1–3). Functional testing is used to evaluate this condition; however, there are no accurate and precise predictive biomarkers. Increased pulmonary artery resistance and pressure are symptoms of PH. The pathophysiological process of PH is characterized by pulmonary artery remodeling, which includes endothelial-to-mesenchymal transition of pulmonary artery endothelial cells and aberrant proliferation of fibroblasts and pulmonary artery smooth muscle cells (PASMCs) (4). The proliferation of PASMCs is the most significant contributor to pulmonary artery remodeling of all contributing elements. Multiple signals, including bone morphogenetic protein signaling, inflammatory signaling, and ion channels, have been linked to the proliferation of PASMCs in PH, according to previous research (5, 6). The prognosis for developing RHF is dismal, and its molecular underpinnings are not fully known due to the intricacy and malignancy of PH. Despite the clinical symptoms of PH having been improved by current medications, the illness is still developing in the majority of patients, and fatality rates are still high (7, 8). Therefore, novel therapeutic and diagnostic modalities are required in clinical settings to promote irreversible pulmonary artery remodeling.

The absence of accurate biomarkers for early PH identification is imperative because PH is typically discovered only in the later stages of the disease, even when signs of RHF manifest. Only proliferating cells typically express the nuclear KI67 protein (9). During interphase, KI67 is largely found in the nucleolar cortex; during mitosis, it is attracted to condensed chromosomes. The two KI67 isoforms, measuring 345 and 395 kDa, are encoded by the KI67 gene, which is found on chromosome 10q25ter. From the G1 phase until mitosis, KI67 expression levels increased before quickly dropping off immediately after mitosis. KI67 protein was not found in G0 quiescent nuclei; however, it was found in mitosis and S, G1, and G2 nuclei. The degree of KI67 expression reflects the stage of cell proliferation (10, 11). KI67 has been suggested to be a prognostic marker for cancer because it is substantially overexpressed in cancer cells (12, 13). MKI67 has been proposed as a potential marker for several cancers and may be linked to the diagnosis of PH (14). Given that it can be detected in the early stages of the disease, KI67 has potential as a prospective biomarker for PH (15). However, how disease progression affects the link to the target organ remains unkown.

Multi-omics analysis has been extensively used to investigate causes of disease since the emergence of high-throughput technologies. Variations in mRNA levels were found by transcriptome profiling. Transcriptome analysis is the basis and starting point for research on gene function and structure. Second-generation high-throughput sequencing allows for accurate and rapid acquisition of nearly all transcript sequence data for a given tissue or organ of a species in a given state (16). It has been extensively employed in various fields including basic research, clinical diagnostics, and medication development.

The present study uses mRNA sequencing to investigate the molecular pathways driving the *in vitro* and *in vivo* onset of PH and to identify the most likely target candidates among them and their functions. These results may point to potential treatment targets and offer molecular proof for evaluating the risk in patients with PH.

## Methods

### Animals and models with monocrotaline

The animal study protocol was reviewed and approved by the Ethics Committee of the Second Xiangya Hospital of Central South University. Male Sprague–Dawley (SD) rats (200–220 g) were purchased from Hunan SJA Laboratory Animal Co., Ltd. (Changsha, China). The Hunan Province, China's Animal Ethics Committee of Central South University, approved all animal studies. One intraperitoneal injection of 50 mg/kg monocrotaline (MCT) (MedChemExpress, Princeton, NJ, USA; cat. #HY- N0750) or 0.9% saline (sham, *n* = 6) was administered to the rats. The rats were euthanized after four weeks, after which the tissue was collected.

### Analysis of echocardiography

Echocardiography is frequently employed as a reliable, non-invasive screening and monitoring method to detect and track the progression of PH. For this, rats that completed the experiment were given isoflurane gas anesthesia and placed supine on a hot plate (37 °C) to avoid hypothermia. A coating of heated ultrasonic gel was applied to the region covering the heart after shaving the chest region to minimize air interference during measurements. A mechanical transducer operating at 10 MHz was used to acquire images in two-dimensional, M-mode, and pulsed Doppler modes. An expert sonographer, who was blinded to the therapy group, conducted all measurements.

### Invasive hemodynamic evaluations

Rats were anesthetized with isoflurane gas following Doppler echocardiographic examination. Forceps were used to grip the animal's paw and quick eye movements were used to gauge the level of anesthesia. Tracheal intubation was performed by placing a polyethylene catheter (PE-50) in the right jugular vein to record the right ventricular systolic pressure (RVSP), which progressed until a sizable positive beat confirmed that it had been positioned in the right ventricle (RV). The catheter was sutured in place and necropsy was performed to determine its location. A pressure transducer connected to a data acquisition system was used to measure the RVSP. The rats were exsanguinated under anesthesia when hemodynamic tests were complete.

### Pulmonary artery remodeling

The right and left pulmonary lobes were longitudinally sectioned, dehydrated, and embedded in paraffin blocks. To qualitatively characterize the vascular remodeling that takes place in the MCT-induced PH model, blocks were sliced into 4-*μ*m thick slices, stained with hematoxylin and eosin (H&E), and inspected using light microscopy by an investigator who was blind to the treatment groups. To gauge the extent of pulmonary vascular remodeling, the percentage of medial wall thickness (% MT) of vessels (50–200 μm diameter) was also examined. A light microscope was used to measure the vessel exterior diameter (ED) and internal diameter (ID) by an observer who was unaware of the animal treatments. To compute MT the following equation was used:

[(ED−ID)/ED]×100

Images were captured using the ImageJ software.

### Construction and sequencing of RNA extraction libraries and tissue RNA extraction

Following the manufacturer's instructions, total RNA was extracted from each mouse cardiac sample by homogenization with TRIzol reagent (Invitrogen, Carlsbad, CA, USA). The volume and purity of each RNA sample were measured. High-quality RNA samples with a RIN > 7.0 were used to build the sequencing library. Total RNA quantity and purity were determined using a Bioanalyzer 2,100 and RNA 6,000 Nano LabChip Kit (Agilent, Santa Clara, CA, USA; cat. #5067–1511). Using Dynabeads Oligo (dT) (Thermo Fisher, Waltham, MA, USA), mRNA was isolated from total RNA (5 μg) after two rounds of purification. Then, a divalent cation and high temperature [Magnesium RNA Fragmentation Module (NEB, Ipswich, MA, USA; cat. #e6150) for 5–7 min], the mRNA was broken up into small fragments. Using *Escherichia coli* DNA polymerase I (NEB; cat. #m0209), RNase H (NEB; cat. #m0297), and dUTP Solution (Thermo Fisher; cat. #R0133), the cleaved RNA fragments were reverse transcribed to produce cDNA by SuperScriptTM II Reverse Transcriptase (Invitrogen; cat. #1896649). The blunt ends of each strand were then given an A-base to ligate them to the indexed adapters. Each adaptor has a T-base overhang to attach to DNA fragments with their A-tailed ends. The fragments were ligated to dual-index adapters and AMPureXP beads were used for size selection. U-labeled second-stranded DNAs was treated with heat-labile UDG enzyme (NEB; cat. #m0280) before the ligated products were amplified using PCR under the following conditions: initial denaturation at 95 °C for 3 min, 8 cycles of denaturation at 98 °C for 15 s, annealing at 60 °C for 15 s, extension at 72 °C for 30 s, and final extension at 72 °C. The resultant cDNA libraries had an average insert size of 30,050 bp. Finally, using an Illumina NovaseqTM 6,000 (LC-Bio Technology Co., Ltd., Hangzhou, China), we carried out 2,150 bp paired-end sequencing (PE150) in accordance with the manufacturer's suggested procedure.

### Functional enrichment analysis and differentially expressed gene (DEG) analysis

The DESeq2 software was used to analyze DEGs between the two groups (and by edgeR between the two groups). The parameters of *q*-value <0.01 and |log2 fold change (FC)| > 1 were used to define DEGs.

Gene Ontology (GO) is a globally accepted system for classifying gene functions. By filtering the DEGs that corresponded to biological functions, GO enrichment analysis revealed that all GO terms were significantly enriched in DEGs compared to the genomic background. First, all DEGs were assigned to GO words in the GO database (http://www.geneontology.org/), and gene numbers were determined for each term. Then, a hypergeometric test was used to identify GO terms that were significantly enriched in DEGs compared to the genome background. Genes frequently interact with each other to contribute to specific biological processes. Pathway-based analyses aid in a greater understanding of the biological operations of genes. The main public pathway-related database is the Kyoto Encyclopedia of Genes and Genomes (KEGG). When compared to the backdrop of the entire genome, DEGs have significantly enriched metabolic or signal transduction pathways according to a pathway enrichment study.

### Data Pre-screening of DEGs using GEO2R and data selection for gene expression profiling

The GSE53408 and GSE113439 Gene expression profiling data were retrieved from the GEO database. The inclusion criteria for gene expression data were as follows: (1) the tested samples were tissues; (2) all tissues had been diagnosed as having PH or were normal tissues; (3) samples were taken from humans; (4) the sequencing chips were the same; and (5) the sample size was >10.

DEGs between the PH and normal samples were examined using GEO2R (http://www.ncbi.nlm.nih.gov/geo/geo2r). Using the limma and GEOquery R packages of the bioconductor project, the web tool GEO2R can be used to compare and evaluate DEGs in MCT lung tissue samples and normal lung tissue samples. To determine the significance of the DEGs, adjusted *p*-value and |log2FC| were employed, and the cut-off conditions were established as |log2FC| > 1 and adjusted *p*-value <0.01.

### DEGs of three distinct samples took the intersection from the Venn diagram

An online platform for bioinformatics analysis called Bioinformatics & Evolutionary Genomics (http://bioinformatics.psb.ugent.be/webtools/Venn/) can be used to examine data. Using the Venn diagram software from this platform, we conducted a cross-analysis of the DEGs of the three independent samples. We examined the DEGs in three distinct samples. The selected genes were then classified as upregulated or downregulated based on the |logFC| values of DEGs in PH tissues compared to in normal tissues in our sequencing data. Venn diagrams were created using evolutionary genetics and bioinformatic tools.

### Protein-protein interaction (PPI) analysis

The STRING database (https://string-db.org) was used to create a (PPI) network of DEGs, which was then displayed using the Cytoscape program (version 3.8.2). The most crucial modules of the PPI network were extracted using the Cytoscape plugin Molecular Complex Detection (MCODE). The top ten hub genes in the entire network were identified using the Cytohubba plugin.

### Transfection of siRNA and cell culture

The lungs of rats were dissected to examine the distal intrapulmonary arteries. Opening the vessel longitudinally, carefully wiping the luminal surface with a cotton swab, and then dissecting it into small pieces allowed the adventitia to be removed and the endothelium to be denuded. They were then put into an upside-down culture flask with DMEM/F12 with 10% FBS at 37 °C and 21% oxygen. After 1 h, the flask was carefully flipped. PASMCs were detected by immunofluorescence labeling using an antibody against smooth muscle *α*-SMA (Abcam, Cambridge, UK; cat. #ab5694) after being seeded in culture dishes and cultivated for 5–7 days in DMEM/F12 with 10% FBS in a humidified environment of 5% CO_2%_ and 95% air at 37 °C. Primary cultures were established, developed to a confluence of approximately 70%, and survived until passages 3–5. For 48 h, PASMCs were subjected to 1% oxygen levels under hypoxia. Cells were transfected with MKI67 siRNA (RiboBio, Guangzhou, Shanghai, China) or an siRNA negative control.

### Quantitative real-time PCR (rt-qPCR)

The GeneJET RNA Purification Kit (Thermo Fisher) was used to extract total RNA from the cell lines in accordance with the manufacturer's instructions. A RevertAid First Strand cDNA Synthesis Kit (Thermo Fisher Scientific) was used to create cDNA according to the manufacturer's instructions. Using PowerUpTM SYBRTM Green Master Mix, RT-qPCR was performed using a Thermo 9,700 Fast Real-Time PCR system (Thermo Fisher). The primer sequences were as follows: GAPDH-F, CAGGAGGCATTGCTGATGAT; GAPDH-R, GAAGGCTGGGGCTCATTT. KIF20B-F, GCCACACCAGTCACAATTAAG; KIF20B-R, ATTTTCACATTTCACCAAGTCCTC. TOP2A-F, AGCAGAAGGTCCCAGAAGAAGAGG; TOP2A-R, AGAAGGTAGTTGAAGGTCGGTCCAG. MKI67-F, TGCTTGCTGGAGTTCTGACTGATTC; MKI67-R, AGCCTGTGGAAGACCTGACTGG. GAPDH served as a control. The 2^−ΔΔCT^ method was used to calculate the fold-change of the RNA.

### Cell counting kit-8 (CCK-8) assay

After receiving the appropriate treatments, PASMCs were seeded in 6-well plates. Each well received 10 μl of CCK-8 reagent (Uelandy, China), which was then incubated for two hours at 37 °C in the dark. The optical density of each sample was measured at 450 nm.

### Wound healing assay

For overnight culture, PASMCs were seeded in 12-well plates. When the cell plate reached 80% confluence, it was scraped vertically using a sterile 10-μl micropipette tip. The serum-free medium was changed after the exfoliated cells were removed using phosphate-buffered saline (PBS) (Thermo Fisher). This was performed for a 48-h incubation. An inverted microscope was used to view and capture cell migration. The test area for wound healing was measured using the ImageJ software.

### Western blot analysis

PBS was used to wash the lungs and PAMSCs. The cells were then lysed using a 1% solution of protease and phosphatase inhibitors in radioimmunoprecipitation assay (RIPA) lysis buffer (Thermo Fisher; cat. #89901 and #78442, respectively). A BCA protein assay kit (Thermo Fisher; cat. #23227) was used to measure the protein concentration in the lysates in accordance with the manufacturer's instructions. Protein samples (30 μg) were separated by 10% sodium dodecyl sulfate-polyacrylamide gel electrophoresis and then transferred onto a polyvinylidene fluoride membrane. Membranes were incubated with the primary antibodies SMAD2/3 (Cell Signaling Technology, Danvers, MA, USA; cat. #8685) and GAPDH overnight at 4 °C after being blocked with 5% non-fat milk for 1.5 h (HyTest, Turku, Finland; cat. #5G4-6C5). The membranes were incubated with an IgG secondary antibody (Abcam; cat. #ab97051) for 1 h at 37 °C the next day, followed by three additional washes with Tris-buffered saline and Tween-20. Antibody-bound proteins were detected using an enhanced chemiluminescence (ECL) reagent (Thermo Fisher; cat. #34580). The grayscale of each band was quantitatively examined using the ImageJ software.

### Statistics analysis

All data are displayed as mean ± standard deviation (SD). GraphPad Prism 8.0 (GraphPad Software, San Diego, CA, USA) was used for all statistical analyses. To evaluate the significance of the differences between the two groups, a two-tailed Student's t-test was used. Statistical analyses were performed using the SPSS software. Statistical significance was set at *p* < 0.05, unless otherwise noted.

## Results

### Establishing rat model of PH

**Figure 1:**
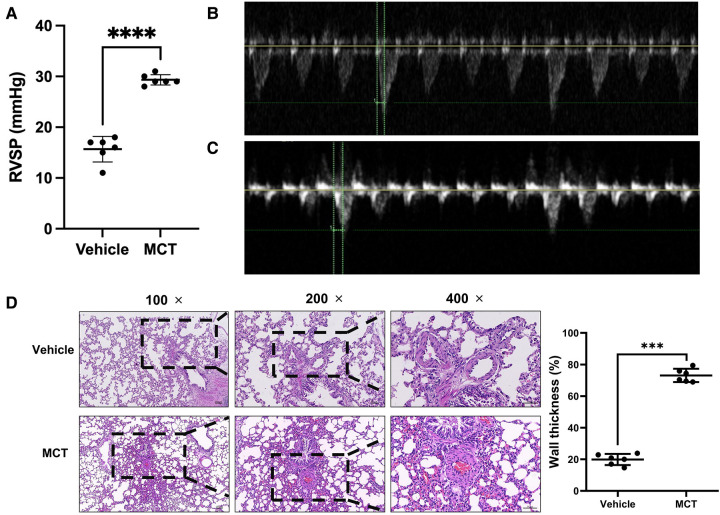
Validation of MCT-constructed PH model. (**A–C**) Cardiac function and RSVP values of rats were measured using echocardiography. (**D**) Lung tissue was stained with H&E to determine the thickness of the blood vessel wall. ***p < 0.001, ****p < 0.0001.

Pulsed Doppler images of the pulmonary artery of MCT-treated rats demonstrated the progression of PH through pulmonary blood flow waveforms. Doppler analysis at the level of the pulmonary valve revealed two separate pulmonary arterial blood flow curves. As evidence of healthy pulmonary vasculature, the pulmonary artery blood flow in the sham group was normal and had a parabolic (symmetry between acceleration and deceleration phases) flow curve (Figure 1B). However, the pulmonary blood flow curve was triangular with notches in MCT rats (Figure 1C). When the rats were administered MCT, their RVSP increased considerably.

RVSP was used to determine whether the patient had pulmonary hypertension. The mean RVSP in the sham group was 15.67 mmHg. MCT rats had a blood pressure reading of 29.33 mmHg. The RVSP in the MCT group was considerably higher than that in the sham group, according to statistical analysis (*p *< 0.0001; Figure 1A). To measure the wall thickness, we removed the lung tissue for H&E staining. The findings revealed that the pulmonary artery wall thickness of MCT-treated rats was dramatically increased (*p* < 0.001; Figure 1D). These findings imply that rats that received MCT treatment had PH.

### DEG profiles

**Figure 2:**
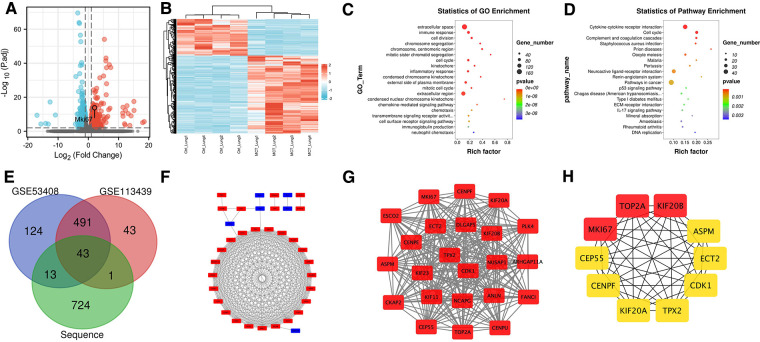
Analysis of sequencing results. (**A**) Volcano map. (**B**) Heat map. (**C**) GO functional enrichment analysis. (**D**) KEGG enrichment analysis. (**E**) Venn diagram. (**F**) PPI network of DEGs. (**G**) the core network calculated by MOCDE. (**H**) Top ten genes calculated by cytoHubba.

Histology and ultrasonography revealed that rat lung tissue that had been exposed to MCT exhibited clear structural and functional alterations. The mRNA expression in the lung tissue of MCT rats and normal rats was compared using RNA-seq technology to clarify the molecular changes and biological processes of lung tissue alteration. A total of 781 DEGs were identified using the selection criteria in the Methods (*q*-value < 0.01 and |FC| > 1), of which 532 were upregulated in MCT-treated lung compared to normal lung and 249 showed downregulation (Figure 2A). DEG expression patterns were shown using heatmaps and grouping of gene expression between MCT-treated and untreated rats was shown by hierarchical clustering analysis (Figure 2B).

### Functional enrichment analysis for DEGs

GO function and KEGG pathway statistical analyses were carried out on the DEGs. Cell division, immune response, extracellular space, chromosomal segregation, centromeric region, mitotic sister chromatid segregation, cell cycle, kinetochore, inflammatory response, and condensed chromosome kinetochore were among the top ten outcomes of GO functional enrichment analysis (Figure 2C). Cytokine-cytokine receptor interaction, cell cycle, complement and coagulation cascades, *Staphylococcus aureus* infection, prion diseases, oocyte meiosis, malaria, pertussis, neuroactive ligand-receptor interaction, and the renin-angiotensin system were the top ten results of KEGG functional enrichment analysis (Figure 2D).

### Identification of DEGs in human PH

After screening the microarray data, DEGs were identified based on the GEO2R analysis (671 for GSE53408 and 578 for GSE113439). According to the Venn diagrams, the three datasets for these DEGs included 43 genes for bioinformatics and evolutionary genomics (Figure 2E). We matched DEGs, including 35 uDEGs and eight dDEGs across PH and normal tissues, to |logFC| values from our sequencing dataset.

### Examination of potential genes in PH

A PPI network was created using the STRING database and Cytoscape software to investigate the biological connections of the DEGs (Figure 2F). The MCODE plugin was used to retrieve a key module from the entire network. Figure 2G demonstrates that this module has 250 edges and 23 nodes (MCODE score = 22.7), most of which are functionally relevant to cell division, such as the regulation of cell cycle pathways. The cytoHubba plugin identified the top ten hub genes, namely, TOP2A, KIF20B, MKI67, CEP55, CENPF, KIF20A, TPX2, CDK1, ECT2, and ASPM (Figure 2H). These may be candidate targets in diagnostic or therapeutic procedures.

### Differentially expressed mRNAs (DEmRNAs) between MCT and normal lung

**Figure 3:**
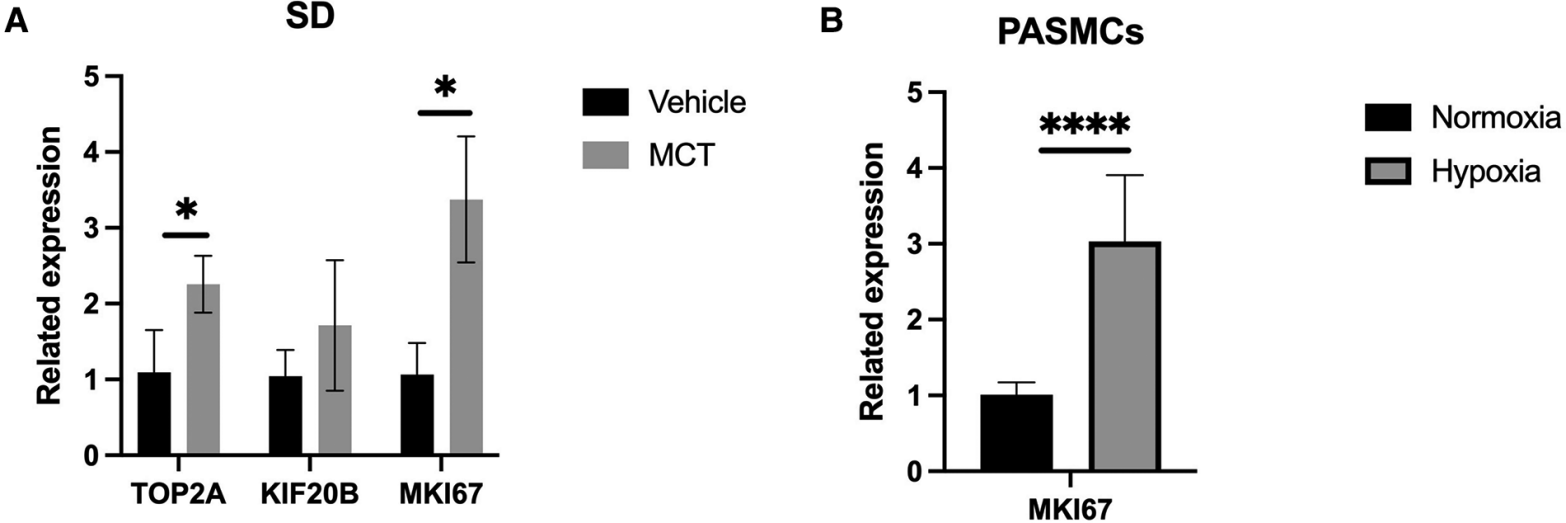
Validation of qPCR. (**A**) qPCR analysis in rat lung tissue. (**B**) qPCR anaysis in construction of hypoxic PASMC model. *p < 0.05, ****p < 0.0001.

We found two DEmRNAs in the lung tissue of MCT-treated and untreated rats (*p* < 0.05). The top three from the top ten were chosen for the experimental validation. RT-qPCR was performed to confirm the expression levels of TOP2A, KIF20B, and MKI67. TOP2A and MKI67 were significantly upregulated in MCT-treated lung tissues compared to control samples, according to the sequencing data (*p* < 0.05; Figure 3A).

### Validation and hypoxia cell model construction

**Figure 4:**
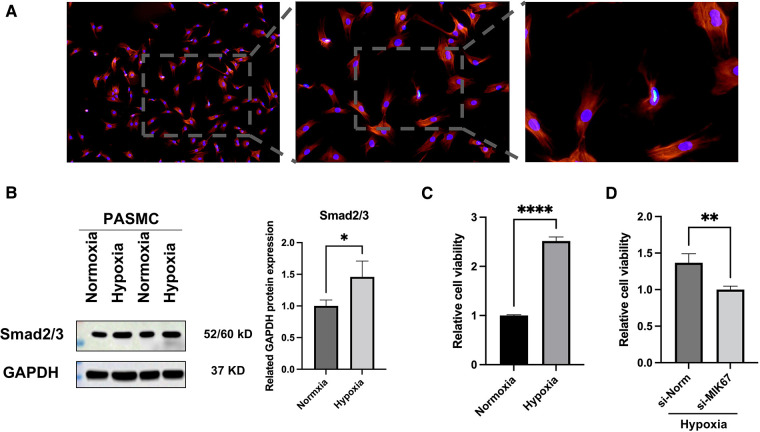
PASMCs and outcomes of hypoxia intervention. (**A**) Immunofluorescence of *α*-SMA. (**B**) Western blot analysis of SMAD2/3. (**C,D**) CCK8 assay. *p < 0.05, **p < 0.01, ****p < 0.0001.

**Figure 5:**
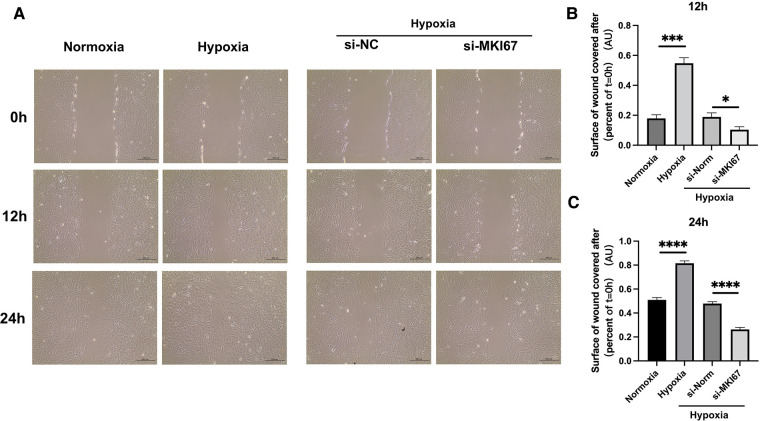
Wound healing assay. ***p < 0.001, ****p < 0.0001.

The accuracy and purity of the PASMCs were confirmed by immunofluorescence. The extracted primary cells were identified as PASMCs using the PASMCs cell-specific marker *α*-SMA. All cells were stained, and no xenogeneic cells were discovered, proving that they were all PASMCs (Figure 4A). Then, a hypoxia model was built, and the marker protein SMAD2/3, wound healing test, and CCK8 studies were utilized to indicate that it enhanced proliferation and promoted migration under hypoxia (Figures 4B,C, 5).

### MKI67 target validation *in vitro*

MKI67, an important mRNA in the mRNA network of MCT-stimulated lung tissues, was found to be related to cell proliferation. To determine whether MIK67 regulates the predicted target genes, MKI67 siRNA was transfected into PASMCs cell lines, which was performed in response to the upregulation of MKI67 expression in a hypoxic environment (Figure 3B). Interestingly, inhibiting MKI67 in PASMCs reversed the effects of hypoxia on cell proliferation and migration, as shown by a reduction in CCK8 proliferation and a decrease in cell migration during the wound healing assay (Figures 4C,D, 5A,C). These findings imply that PASMC migration and proliferation are inhibited by MKI67 suppression in hypoxic environments.

## Discussion

A mean pulmonary arterial pressure >20 mmHg and a pulmonary vascular resistance of precapillary lesions of three or more Wood units are now considered to be symptoms of PH. A frequent mechanism by which increased pulmonary artery pressure causes increased right pulmonary artery pressure is RV afterload, one of the numerous etiologies of the condition put into the World Health Organization group categorization system (17). RHF may develop because of a variety of mechanical and biochemical changes brought on by this increase in afterload, both adaptive and maladaptive. When PH is present, RHF is characterized by an elevated afterload that causes RV dysfunction, resulting in a syndrome with heart failure-related clinical signs and symptoms (18). In PH, hemodynamic instability, cardiogenic shock, and more protracted symptoms that worsen over several months can all present promptly with RHF. Chronic RV afterload increases cause chronic RHF because they eventually exceed the RV compensating mechanisms (19). RHF causes symptoms linked to decreased cardiac output and venous congestion throughout the body. Despite the difficulty in determining the exact incidence of RHF in PH due to the lack of a strict definition of RHF, it is the primary cause of death from PH. With a baseline five-year survival rate of only 57%, RV dysfunction appears to be a powerful predictor of poor clinical outcomes in PH (20, 21). RV dysfunction has no unique laboratory indicators; hence, it is ideal to identify biomarkers during PH for early diagnosis and treatment.

Multiple pathogenic or hereditary factors contribute to the gradual, complicated, and destructive nature of PH. RHF, which has a significant influence on people's lives, can result from end-stage diseases (3, 22). One of the primary characteristics of PH is abnormal pulmonary arterial remodeling. The proliferation and migration of pulmonary artery smooth muscle cells have increased, which has a tumor-like effect on the pulmonary artery remodeling process (5, 23). The intricacy and malignancy of PH make it difficult to fully understand the molecular pathways, even though earlier research have demonstrated that various signaling contributes to PASMCs proliferation and migration in PH. Finding new targets can have a significant impact on how well we comprehend the pathophysiology of PH and how to diagnose and treat PH. In the current study, we showed that MKI67, a protein that influences cell proliferation, facilitates PASMC proliferation and migration brought on by hypoxia.

KI67 is a significant proliferation marker used in pathology (24). Monoclonal antibodies initially identified KI67 as an antigen in the nuclei of developing cells (25). Even when antibodies against KI67 are used to diagnose cancer, the function of the protein remains unknown. The only significant characteristic of KI67 is its absence from resting cells and its expression during cell growth (26). The KI67 labeling index has evolved into a standard for the diagnosis and prognostic evaluation of cancer patients since it was first developed (27). Despite the importance of these findings for illness diagnosis, results on KI67 function have recently been published. KI67 can be used as a general marker for diagnosis and prognosis when combined with cancer tissue-specific markers.

There is little information regarding MKI67 gene expression compared to what it known about the significance of KI67 protein expression for cancer diagnosis. It has been established that the cell cycle regulates the expression of KI67 mRNA and that the KI67 protein is destroyed by the proteasome during the G phase and at cell cycle exit (28). According to recent studies, DNA damage induction, p53 activation, and cell cycle regulation of MKI67 gene expression and KI67 protein synthesis work together to control these processes (29). The main pathogenic change associated with PH is increased growth of muscle cells. MKI67 is not a molecule involved in control from molecular biology studies in PH. MKI67 transfection was used in this investigation to demonstrate how MKI67 affects cell proliferation and migration in the pathogenesis of PH. MKI67 is an effective indicator for PH diagnosis and management.

Several detection techniques were employed in the present study to verify that an animal model of PH caused by MCT was successfully created. Next, we used RNA-seq technology to sequence the excised lung tissue. Bioinformatic technology was used to analyze and use the data. We compared our sequencing data with data from open databases to rule out potential targets, which increased the accuracy of the data. Following the experimental validation of the top three targets, we chose MKI67 as the research target because it had the most pronounced differential expression. A cellular model of PH was created and its effectiveness was evaluated. MKI67, a crucial element of the cell cycle that regulates PASMC proliferation, was enhanced in PASMCs subjected to hypoxia. Thus, we suggest that MKI67 plays a role in the regulation of hypoxia in PASMCs. Notably, our results showed that hypoxia significantly increased PASMC proliferation. This hyperproliferation and migration were reversed when MKI67 was knocked down by siRNA transfection, suggesting that MKI67 was responsible for this function.

Our data intersected with PH samples from the other two datasets, which greatly improved the reliability and accuracy of the results. Out of the three study objectives with the highest scores, we chose the one with the largest difference in the testing outcomes. MKI67 is a cell proliferation marker that has been applied to the study of cancer. Although KI67 has been utilized as an indicator in numerous investigations of PH, no study has examined whether it plays a regulatory role in this condition. Our research shows that MKI67 is both a viable biomarker and a possible therapeutic target for PH.

The current study had several limitations. First, although the downstream pathway of MKI67 will be the focus of our future research, this pathway was not examined further here. Second, we did not conduct animal rescue studies or test the hypothesis that MKI67 inhibition could reverse the PH phenotype in living organisms. To achieve better outcomes, we plan to conduct an in-depth study in the future.

The present study revealed that the proliferation and migration of PH and PASMCs are caused by hypoxia, which is mediated by MKI67. Taken together, MKI67 may be a promising target for the detection and therapy of PH.

## Data Availability

The datasets presented in this study can be found in online repositories. The names of the repository/repositories and accession number(s) can be found below: NCBI Short Read Archive (SRA) with accession number with Accession: PRJNA885779.
